# Dental Convention

**Published:** 1874-11

**Authors:** A. S. Billings

**Affiliations:** Sec. and Treas.


### DENTAL CONVENTION.

The Missouri Valley Dental Society met in annual session at the office of Dr. F. M. Shriver, of Glenwood, Ia., July 22d and 23d. President Dr. Billings in the chair.

Minutes of last meeting read and approved.

After the admission of new members, work was at once commenced.

An essay was delivered by Dr. J. F. Sanborn, entitled "Dentistry of twenty-five years past compared with the present."

Discussion by Dr. C. Thomas and others.

The committee appointed on the Barnum Testimonial Fund reported in favor of paying $10 out of the funds, and the treasurer was ordered to do so.

Dr F. M. Shriver showed some very beautiful specimens of combined gold and rubber plate work (in actual use).

Dr. A. S. Billings gave them an opportunity of seeing the working of the Morrison engine.

All present seemed deeply interested in the discussions and proceedings, and declared it one of the best meetings we have ever had.

The following committees were elected:

Ex. Committee, Dr. C. Thomas, J. W. Chadwick, of Nebraska City; Dr. Sanborn, Tabor, Iowa.

Membership Committee, Drs. Sanborn, Shriver, Woodbury.

The retiring President gave an essay on the treatment of devitalized nerve and pulp cavity for filling.

The election of officers for the ensuing year resulted as follows:

President, Dr. C. Thomas; Vice President, F. M. Shriver; Secretary and Treasurer, Dr. A. S. Billings.

On motion, adjourned to meet in Nebraska City (at the office of Dr. Thomas) on the fourth Tuesday in July, 1875.

A. S. Billings, Sec. and Treas,

